# A low-cost, LED based, fluorescent antibody test for the detection of the five human malaria species

**DOI:** 10.1186/1475-2875-9-S2-P40

**Published:** 2010-10-20

**Authors:** G-Halli R Rajasekariah, Diane Dogcio, Rogan Lee, Bernard J Hudson, Hubert G Mazure, Anthony M Smithyman

**Affiliations:** 1 Cellabs Pty Ltd 7/27 Dale St Brookvale NSW 2100 Australia; 2 ICPMR, Westmead Hospital, Westmead, NSW, Australia; 3 Department of Microbiology, Royal North Shore Hospital, St Leonards, NSW Australia

## Introduction

Conventional blood film microscopy for malaria diagnosis remains a tedious and time-consuming activity requiring examination of 100 to 200 Giemsa stained oil-immersion fields. In expert hands, blood film microscopy is highly sensitive but for the vast majority of laboratories accuracy of diagnosis is little better than 70%, particularly at low levels of parasitaemia. The introduction of lateral flow immunochromatography rapid tests (RDTs) over the past 15 years has gone some way towards addressing this problem but again these tests encounter sensitivity problems particularly when parasite numbers are low. The issue of sensitivity can be overcome by multiplex PCR assay systems but at the present time this technology is restricted to a few major reference laboratories. One sensitive immunological method that has not been applied to malaria diagnosis is direct immunofluorescence (DFA), mainly because the high cost and maintenance of UV microscopes has made their routine use impractical and prohibitively expensive. However the availability of a new generation of low cost, LED fluorescence microscopes (powered by mains, car battery or solar battery) puts this technology in reach of almost any laboratory or field station. One LED adapter allows the conversion of any light microscope into a fluorescent microscope in a matter of minutes. We report here the development of a DFA staining technique for the detection of the five human malaria species. The method is highly sensitive with antibody specificity providing a significant advantage to the operator.

## Methodology and preliminary findings

**Figure 1:**
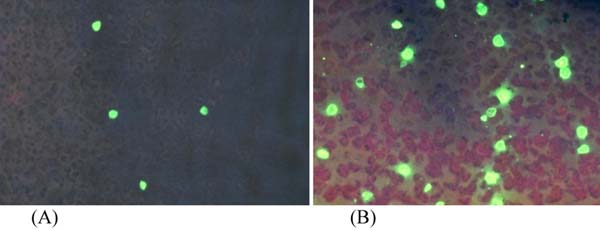
Pictures of iRBCs (magnification 400x) in thin smears. prepared from (A) 0.01% *P. falciparum* positive blood sample and (B) >1% P. falciparum positive sample, stained by direct IF reagent and visualized under x40 objective (400x magnification).

The test is simple to perform, requiring only 30 min for completion. In brief, thin blood smears are prepared using 2 to 4 μl of blood, air dried, fixed in methanol and stained with DFA reagent for 30 min. After mounting, the stained slides are examined under the LED UV microscope. By scanning as few as 10 microscopic fields, indicative test results can be obtained. The overall testing procedure requires less than one hour. Malaria-infected red blood cells appear as bright apple-green "beacons" against a dark reddish-brown background. This reagent can be used to detect parasitaemias ranging from as low as <0.01% to as high as 9% of infected RBCs (iRBCs). Stained iRBCs containing parasites can easily be seen under a x40 objective lens with x10 eye piece as shown in Figure 1. The method is highly sensitive and can detect as low as 2 to 4 parasites per microlitre (μl) of blood. This method has been used for detection of the five human malaria (*P. falciparum, P. vivax, P. ovale, P. malariae, P. knowlesi*) parasites (Table 1). One major advantage of this technique is the ability to detect malaria infection in low-parasitaemia samples even at 400x magnification (using x40 objective and x10 eye piece). Thus it is possible to scan a greater number of fields in far less time with a significant drop in operator fatigue.

**Table 1:** 

Parasitaemia	Stained with	Parasites/micro-field (400x magnification)	Mean±SD
P. falciparum (0.01%)	Pf-FITC	0,0,1,1,2,2,2,	1.1 ±0.8
P. falciparum (0.1%)	Pf-FITC	0,1,2,3,4,5,6,	3.0±2.1
P. falciparum (0.7%)	Pf-FITC	5,7,10,12,13,16,22	12.1 ±5.6
P. falciparum (1.4%)	Pf-FITC	Too many to count	ND
P. vivax	Plasm-FITC	0,0,0,1,1,1,2	0.7±0.7
P. ovale	Plasm-FITC	1,2,4,4,6,8	4.2±2.5
P. knowlesi	Plasm-FITC	9,11,12,13,15	12±2.2

## Conclusions

Direct fluorescent antibody staining of malaria parasites coupled with the use of low cost LED microscopes should increase the reliability of malaria diagnosis in resource-deprived settings and provide enhanced sensitivity, thus facilitating reliable detection of low density parasitaemias and subsequent early treatment, which may aid in minimizing emergence of drug resistance.

